# Evaluating Surveillance Strategies for the Early Detection of Low Pathogenicity Avian Influenza Infections

**DOI:** 10.1371/journal.pone.0035956

**Published:** 2012-04-24

**Authors:** Arianna Comin, Arjan Stegeman, Stefano Marangon, Don Klinkenberg

**Affiliations:** 1 Istituto Zooprofilattico Sperimentale delle Venezie, Legnaro, Padua, Italy; 2 Department of Farm Animal Health, Faculty of Veterinary Medicine, Utrecht University, Utrecht, The Netherlands; University of Hong Kong, Hong Kong

## Abstract

In recent years, the early detection of low pathogenicity avian influenza (LPAI) viruses in poultry has become increasingly important, given their potential to mutate into highly pathogenic viruses. However, evaluations of LPAI surveillance have mainly focused on prevalence and not on the ability to act as an early warning system. We used a simulation model based on data from Italian LPAI epidemics in turkeys to evaluate different surveillance strategies in terms of their performance as early warning systems. The strategies differed in terms of sample size, sampling frequency, diagnostic tests, and whether or not active surveillance (i.e., routine laboratory testing of farms) was performed, and were also tested under different epidemiological scenarios. We compared surveillance strategies by simulating within-farm outbreaks. The output measures were the proportion of infected farms that are detected and the farm reproduction number (R_h_). The first one provides an indication of the sensitivity of the surveillance system to detect within-farm infections, whereas R_h_ reflects the effectiveness of outbreak detection (i.e., if detection occurs soon enough to bring an epidemic under control). Increasing the sampling *frequency* was the most effective means of improving the timeliness of detection (i.e., it occurs earlier), whereas increasing the sample *size* increased the likelihood of detection. Surveillance was only effective in preventing an epidemic if actions were taken within two days of sampling. The strategies were not affected by the quality of the diagnostic test, although performing both serological and virological assays increased the sensitivity of active surveillance. Early detection of LPAI outbreaks in turkeys can be achieved by increasing the sampling frequency for active surveillance, though very frequent sampling may not be sustainable in the long term. We suggest that, when no LPAI virus is circulating yet and there is a low risk of virus introduction, a less frequent sampling approach might be admitted, provided that the surveillance is intensified as soon as the first outbreak is detected.

## Introduction

The surveillance and control of avian influenza (AI) have historically focused on the detection and eradication of infections with highly pathogenic avian influenza (HPAI) viruses in poultry populations. However, since low pathogenicity AI (LPAI) viruses of the H5 and H7 subtypes can mutate into HPAI viruses, as occurred for example in 1999 in Italy [1] and in 2003 in Chile [2], the detection and control of LPAI has become compulsory in the European Union (EU) [3]. Moreover, in 2008, the rapid mutation of an LPAI H7N7 virus strain (2–3 weeks after its introduction) that resulted in the HPAI outbreak in the United Kingdom and evidence of multiple incursions of different AI viruses in EU in recent years have highlighted the need for early detection [4].

For HPAI, early detection has been successfully based on passive surveillance (i.e., the observation of signs and symptoms), given that infection induces clear clinical signs and high mortality in most poultry species. However, for LPAI, the signs may go unnoticed by passive surveillance and mortality is low [5]. For this reason, active surveillance is performed (i.e., visiting farms and sampling animals for diagnostic tests) [6].

In the EU, current AI surveillance focuses on determining the presence of infections with subtypes H5 and H7 in different poultry species [3], [6]. To this end, sampling strategies have been set up to detect a fixed design prevalence with a certain probability (i.e., assuming minimum detectable prevalences (i.e., design prevalences) of 5% LPAI infected holdings and 30% infected animals within an infected holding). However, these methods neglect the dynamics of the infection in the population and may result in missed or delayed detection [4].

Over the years, both the number of EU Member States that have implemented AI surveillance and the number of samples tested have increased. However, evaluations of the effectiveness of LPAI surveillance programs have mainly focused on their performance in establishing the presence of infected birds or assessing freedom from infection [7], [8] and not on their performance as early warning systems for new introductions. For this reason, an optimal design for early warning surveillance has not been defined.

Italy is the EU country with the largest number of AI outbreaks, and it was the first to implement an AI surveillance system, which also consisted of the routine laboratory testing of poultry farms [9]. Since the system was made operational, four major LPAI epidemics have occurred, in 2000–2001, 2002–2003, 2004, and 2005, in addition to two small epidemics in 2007 and 2009, which mainly involved non-industrial flocks. Given that this is the longest running surveillance system in Europe, a large quantity of data has been collected, which, together with the experience gained, could be useful in evaluating and improving surveillance programmes. In fact, in a previous study [10] we investigated the transmission dynamics of LPAI within turkey farms using serosurveillance data from the above epidemics and experimental infection data. That study showed that the basic reproduction number (R_0_) within farms was on average 5.5, meaning that an infectious turkey infects on average 5.5 turkeys within a susceptible farm, although R_0_ varied greatly among farms. Furthermore it resulted in final estimates for the mean latent and infectious periods of 2.9 and 8.1 days, respectively, and provided a mathematical model to simulate within-farm outbreaks of LPAI.

The objective of the present study was to evaluate different surveillance strategies in terms of their capacity to act as early warning systems for LPAI epidemics in an area with a high risk of virus exposure. To this end, we carried out simulations with a model parameterized with data from the Italian LPAI epidemics [10]. Although future outbreaks will probably be with different strains and in different areas, this is the model that combines most available field data and therefore represents the best available knowledge. The strategies differed in terms of the number of samples collected, sampling frequency, the type of laboratory tests performed, whether or not active surveillance was performed, and when active surveillance was begun. Our simulations were based on actual data and were performed considering turkeys, which has been the most affected species in Italy.

## Methods

We compared surveillance strategies by simulating within-farm outbreaks. As output measures, we used the proportion of infected farms (i.e. outbreaks) that are detected and the farm reproduction number (R_h_) (i.e., the average number of outbreaks caused by one infectious farm) of an infected farm at detection, assuming that the farm is not infectious to other farms after detection (e.g., because of culling or quarantine). The proportion of infected farms that are detected provides an indication of the sensitivity of the surveillance system to detect within-farm infections. It is a result of surveillance that is easier observed in the field but has no direct relation to R_h,_ which, in turn, reflects the effectiveness of outbreak detection: an R_h_ of <1 at detection (i.e., the threshold value to prevent an epidemic) means that the infection is detected (and stopped) at an early stage, so that epidemics can be brought under control.

### Within-farm surveillance model

Within-farm outbreaks were simulated by means of the stochastic susceptible-exposed-infectious-recovered (SEIR) model (baseline model) of Comin *et al.* [10]. The parameters are given in Table 1. Farms were assumed to consist of a single flock. Outbreaks started at a uniform random flock age with one latently infected bird. We assumed that the latent and infectious periods had a gamma distribution, with average durations of 2.9 and 8.1 days, respectively [10]. During their infectious period, birds transmitted the virus to susceptible birds within the farm at rate β, which was chosen such that the mean number of secondary cases generated by one infectious bird (i.e., R_0_) is equal to 5.5 [10]. Simulations were carried out using *R* [11].

**Table 1:** Parameters used in the within-farm transmission model (from Comin *et al* [10]).

	value in the baseline model	parameters' value
time step	0.02 days	-
simulation period	130 days	-
farm size	10,000 turkeys	-
day of virus introduction	∼uniform(a; b)	a = 0; b = 130
farm-specific basic reproduction number	∼gamma(s; r)§	s = 2.73; r = 0.49
bird-specific duration of latent period	∼gamma(s_L_; r_L_)	s_L_ = 17.41; r_L_ = 5.95
bird-specific duration of infectious period	∼gamma(s_I_; r_I_)	s_I_ = 4.64; r_I_ = 0.57

**Footnote:**

§ s = shape parameter; r = rate parameter.

Detection may take place by passive or active surveillance. For passive surveillance, we assumed that detection occurs when the proportion of infected birds was sufficient for allowing clinical disease to be detected in the farm, which occurred when the within-farm prevalence reached the detection threshold, D (see below for estimation of D). For active surveillance, detection could occur on specific sampling days, depending on the surveillance strategy (e.g., 60, 90, and 120 days after the onset of the production cycle in the reference strategy). On a given sampling day, a random sample of birds was taken, and if at least one of them was infectious (i.e., positive virological test) or had recovered from infection (i.e., positive serological test), then the sampling day was defined as detection day.

**Figure 1:**
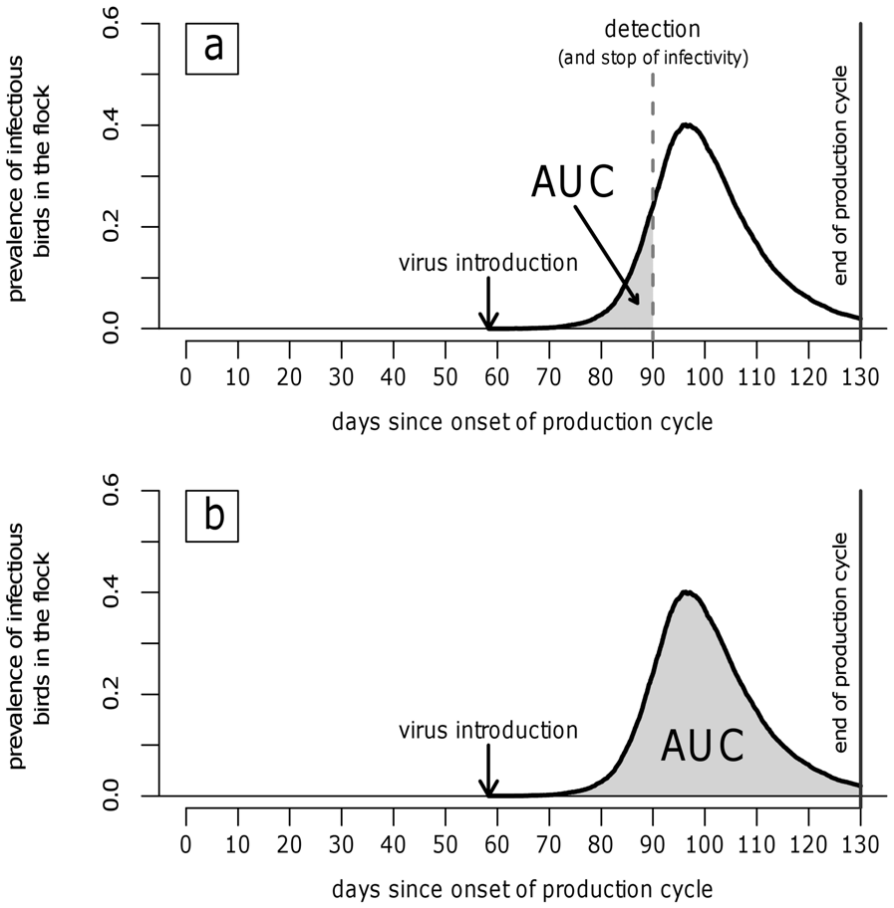
Area under the prevalence curve (AUC, shaded area). (a) AUC assuming that the infectivity is efficiently stopped at day of outbreak detection. (b) AUC in the whole infectious period.

Once an infected farm was detected, the expected number of farms infected secondarily prior to detection was calculated by multiplying the bird-to-farm transmission rate (β_h_, the average number of farms infected by one infected bird per unit of time) by the cumulative number of infectious birds (area under the curve, AUC) up to the detection day. If an outbreak was still not detected on the last sampling day, the AUC at the time of slaughter was used (Figure 1.b). By repeating this for many simulated outbreaks, the mean of β_h_*AUC was computed, which is interpreted as the between-farm reproduction number R_h_ (i.e., the mean number of farms infected by a single infected farm).

### Estimation of D, the detection threshold for passive surveillance

To estimate D, we used the field data provided by the intensive surveillance system in place in Italy during the LPAI epidemics, which is described in detail elsewhere [9]. During and around the time of the epidemics, a total of 6,102 poultry farms were routinely visited; there were more than 13,000 sampling events, and 497 outbreaks (i.e., infected farms) were detected. For the calculation of D, we used data from only unvaccinated infected turkey farms with multiple samplings and available information on farm size, mean sampling interval, mean sample size and whether the outbreak had been detected by active or passive surveillance. Because many records were incomplete, we also included farms consisting of multiple flocks to increase the number of data points. As outbreaks on farms generally start in one flock and will be detected in that flock, we believe that inclusion of these multiple-flock farms is valid. Based on these criteria, 96 turkeys farms were selected: 72 (75%) were detected by active surveillance and 24 (25%) by passive surveillance. Unfortunately, we could not directly use the data from the 24 flocks detected by passive surveillance, because they did not contain any information about the prevalence at the time of clinical suspicion, since sample collections are always performed some days later, after farmer's notification. As an alternative, we assumed that passive detection is related to the prevalence of infectious birds, as that probably best reflects the clinical picture on the farm. The prevalence threshold for passive detection, D, was thus determined by first simulating outbreaks with flock sizes and active surveillance scheme as in the dataset, and then looking what threshold prevalence would have resulted in 24 farms being detected by passive rather than active surveillance (as in the dataset).

Following this assumption, to estimate D we simulated 100 sets of 96 outbreaks, using the average flock sizes reported in the aforesaid dataset and the corresponding 96 farm-specific sampling intervals and sample sizes (Table 2). This resulted in 96 detection times for each set of simulations and 96 peak prevalences at or before the day of active detection. In order to have 24/96 outbreaks detected by passive rather than active surveillance (as in the data), the minimum detectable prevalence was selected as the 24^th^ highest peak prevalence. In brief, we performed the following steps:

1. simulate 96 outbreaks, with flock size, sampling interval, and sample size being the same as in the data
2. determine the maximum of all prevalences until the day of detection for each outbreak
3. arrange the maximum prevalences in order and select the 24^th^ highest as the minimum detectable prevalence
4. repeat 1–3 100 times to have 100 minimum detectable prevalences
5. determine mean and 95% confidence interval

**Table 2:** Descriptive statistics of the farm characteristics and surveillance schemes in the 96 turkey farms selected to estimate the detection threshold for passive surveillance.

	mean	SD	5^th^ percentile	95^th^ percentile
number of birds per farm	14 000	7856	5075	26 825
number of flocks per farm	2.2	1.5	1	5
number of birds per flock	8139	6496	2317	18 050
duration of the production cycle (days)	138	22	98	163
day of first sampling	65.1	22.6	29.0	103.0
day of detection	109	23	65	141
number of sampling events per farm	3.2	1.2	2.0	5.3
average sampling interval per farm	23.5	12.1	10.2	49.5
average number of samples collected per flock	6.2	3.3	2.3	10

### Estimation of β_h_

The bird-to-farm transmission rate β_h_ represents the number of newly infected farms generated by a single infectious bird housed in an infected farm per unit of time. For estimation of β_h_, the relation R_h_ = β_h_*AUC can be reversed for a known value R_h_' during an epidemic, and a mean AUC' of actual flocks during that same epidemic: β_h_ = R_h_'/AUC'. For this calculation, we used a published value of R_h_' of 2.15, which was the farm reproduction number at the beginning of the Italian 2000–2001 LPAI epidemic, when no control measures were in place yet [12]. The value of AUC' was estimated as the average AUC of 1000 simulated outbreaks in 1000 single-flock farms with different flock sizes and gender of birds (randomly selected from a list of single-flock turkey farms available at the Regional poultry farm registry), assuming the absence of any control measure. For each outbreak, we simulated the day of virus introduction (from day 0 to 100 or 130 of the production cycle, depending on the gender of the turkeys) and the infection dynamics, using the above-described SEIR model and parameters. We assumed that the infection process in a farm continued until it reached its end or the production cycle was stopped (i.e., because control measures were not in place) (Figure 1.b). After repeating the simulation 1000 times, we calculated the mean and 95% confidence interval of the AUC in absence of control measures.

### Simulation of surveillance strategies

To simulate different surveillance strategies, we combined the information on within- and between-farm disease dynamics, the number of samples, the frequency of sampling, and the type of surveillance (passive, active, or both, and whether virological or serological testing was performed), and the detection ability of passive surveillance, assuming that a farm stopped being infective immediately after detection.

In the reference surveillance strategy, we assumed a combination of both active and passive surveillance applied to a single-flock farm of 10,000 male turkeys. Sampling for active surveillance was assumed to start at day 60 of the production cycle (based on the results of Comin *et al.* [9]) and to be performed on a monthly basis (as in Mulatti *et al.* [12]). At each sampling, we assumed that 10 birds were tested by means of both serological and virological assays (assuming perfect accuracy for both tests, which was relaxed in sensitivity analyses, see below). If the sample included at least one positive bird, the farm was considered to be infected and the corresponding sampling day was defined as the detection day. However, if the prevalence of infectious birds had exceeded the threshold D before positive samples were found, we assumed that the infection had been found by passive surveillance and the first day on which the prevalence exceeded the threshold was considered as the detection day.

The reference strategy was compared to six alternatives, namely: starting the active surveillance at day 30 of the production cycle, increasing the sampling frequency to once every 15 days, decreasing the sampling frequency to once every 60 days, collecting 30 samples per sampling, performing only serological testing, and applying only passive surveillance (Table 3). For each surveillance strategy we calculated the AUC at detection day (i.e., the area under the prevalence curve until detection day) (Figure 1.a) and at different time points after detection day (i.e., from 1 to 10 days), given that in reality culling is not performed on detection day. The farm reproduction number of an infected farm at the different time points was calculated by multiplying the AUCs by β_h_.

**Table 3:** Summary of the investigated surveillance strategies.

Surveillance strategy:	sampling days	surveillance components^a^	# samples per sampling	tests^b^
reference	60–90–120	AS+PS	10	ST+VT
start day 30	30–60–90–120	AS+PS	10	ST+VT
freq 15 days	60–75–90–105–120	AS+PS	10	ST+VT
freq 60 days	60–120	AS+PS	10	ST+VT
30 samples	60–90–120	AS+PS	30	ST+VT
only serology	60–90–120	AS+PS	10	ST only
only PS	-	only PS	-	-

**Footnote:**

a AS = active surveillance; PS = passive surveillance.

b ST = serological testing; VT = virological testing.

### Sensitivity analysis

The simulated surveillance strategies referred to the Italian situation at the beginning of the 2000–2001 LPAI epidemic, when the first outbreaks appeared and no control measures were in place. To investigate how the surveillance strategies would perform under different conditions, we simulated some alternative scenarios (Table 4), exploring adjustment of the strategy by varying parameters one by one. In particular we assumed:

- imperfect sensitivity (Se) of serological testing;
- imperfect sensitivity of virological testing;
- larger farm size;
- smaller farm size;
- earlier virus introduction;
- later virus introduction;
- longer mean generation time (i.e., generation time is defined as the mean time interval between infection of a primary case and infection of secondary cases caused by the primary case);
- shorter mean generation time;
- higher mean basic reproduction number;
- lower mean basic reproduction number.

**Table 4:** Summary of the alternative scenarios investigated in the sensitivity analysis.

Reference settings	mean R_0_	mean generation time (days)	farm size (turkeys)	serological test Se	virological test Se	mean virus introduction (days)
	5.55*	7.90*	10000	100%	100%	54.71
Lower serological test sensitivity	Sensitivity of serological test = **90%** §					
Lower virological test sensitivity	Sensitivity of virological test = **85%** §					
Larger farm size	**20000** turkeys in the farm					
Smaller farm size	**5000** turkeys in the farm					
Earlier virus introduction	Virus introduction: on average **27.18** days after the onset of the production cycle [introduction day∼uniform(a; 54.71)]					
Later virus introduction	Virus introduction: on average **80.94** days after the onset of the production cycle [introduction day∼uniform(54.71; b)]					
Longer generation time	Mean generation time: **11.85** days [LP∼gamma(s_L_; r_L_/1.5); IP∼gamma(s_I_; r_I_/1.5)]					
Shorter generation time	Mean generation time: **5.27** days [LP∼gamma(s_L_; r_L_ *1.5); IP∼gamma(s_I_; r_I_ *1.5)]					
Higher R_0_	Mean basic reproduction number: **11.19** [R_0_∼gamma(s; r/2)]					
Lower R_0_	Mean basic reproduction number: **3.11** [R_0_∼gamma(s; r*2)]					

* values derived from Comin *et al.* [10].

§ values based on van der Goot *et al.* [13].

**Footnote:** values of parameters a, b, s_L_, r_L,_ s_I_, r_I_, s and r are those previously reported in Table 1.

## Results

The average threshold prevalence for outbreak detection by passive surveillance was 56.0% (95%CI: 55.5–56.5%), meaning that on average roughly half of the birds in the farm would have to be simultaneously infected (i.e., virus-positive) for LPAI infection to be clinically suspected. The estimated mean AUC at the end of the outbreaks was 57,061 birds (95%CI: 54,162–59,960), which, when divided into R_h_ in the absence of control measures [12], yields the number of farms that one infectious bird can infect per day: β_h_ = 3.768·10^−5^ (95%CI: 3.586·10^−5^–3.970·10^−5^).

**Figure 2:**
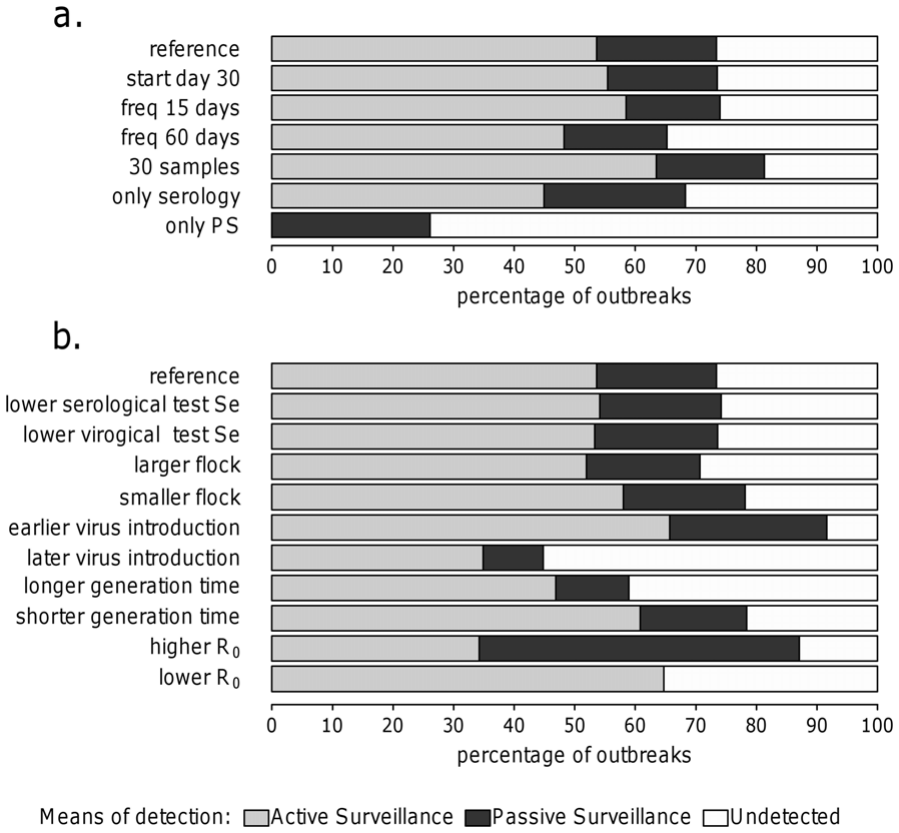
Percentages of outbreaks detected by active and passive surveillance. (a) Various surveillance schemes under the reference scenario. (b) Reference surveillance scheme under different scenarios (i.e., sensitivity analysis). **Footnote**: Se = sensitivity.

Table 5 and Figure 2.a summarize the results of the surveillance strategies under the reference epidemiological scenario (i.e., early incursions of LPAI viruses in turkey farms in the absence of control measures). When considering the reference surveillance strategy, 73% of the infected farms were detected, on average 35 days after virus introduction and mainly by active surveillance (54% of infected farms, 74% of detected outbreaks). The simulation of alternative surveillance strategies suggested that decreasing the sampling frequency from once a month to once every two months leads to detection on average 43 days after virus introduction, and performing only passive surveillance reduces the detection rate to 26% of infected farms, thus making it impossible to detect the infection soon enough for avoiding the spread of the virus to other farms (i.e. R_h_>1).

**Table 5:** Detection ability towards LPAI infections in absence of control measures (reference scenario).

Surveillance model	proportion of outbreaks that are detected	proportion of outbreaks that are detected by active surveillance	mean detection time since virus introduction (days)	mean prevalence in the farm at detection	R_h_ at detection [95%CI]
reference	73.4%	53.6%	34.9	31.3%	0.80 [0.75–0.85]
start day 30	73.5%	55.4%	34.5	30.6%	0.77 [0.72–0.82]
freq 15 days	74.0%	58.5%	30.8	27.7%	0.53 [0.49–0.57]
freq 60 days	65.2%	48.2%	43.2	27.6%	1.21 [1.14–1.28]
30 samples	81.3%	63.5%	34.9	26.5%	0.62 [0.57–0.66]
only serology	68.3%	44.9%	36.5	34.5%	1.05 [0.99–1.11]
only PS	26.1%	–	22.4	59.2%	1.48 [1.41–1.55]

Results of 1000 simulated outbreaks applying different surveillance strategies.

The detection rate of the reference surveillance strategy under different scenarios (sensitivity analysis) is shown in Figure 2.b. The use of imperfect diagnostic tests (90% and 85% sensitivity for serological and virological testing, respectively) did not result in a significant decrease in the percentage of detected outbreaks. Furthermore, surveillance was more sensitive in smaller farms. The reference scenario refers to the LPAI virus that was introduced in Italian farms at the beginning of the LPAI epidemic in 2000; if a strain with a higher R_0_ was introduced, the role of passive surveillance would seemingly be enhanced.

**Figure 3:**
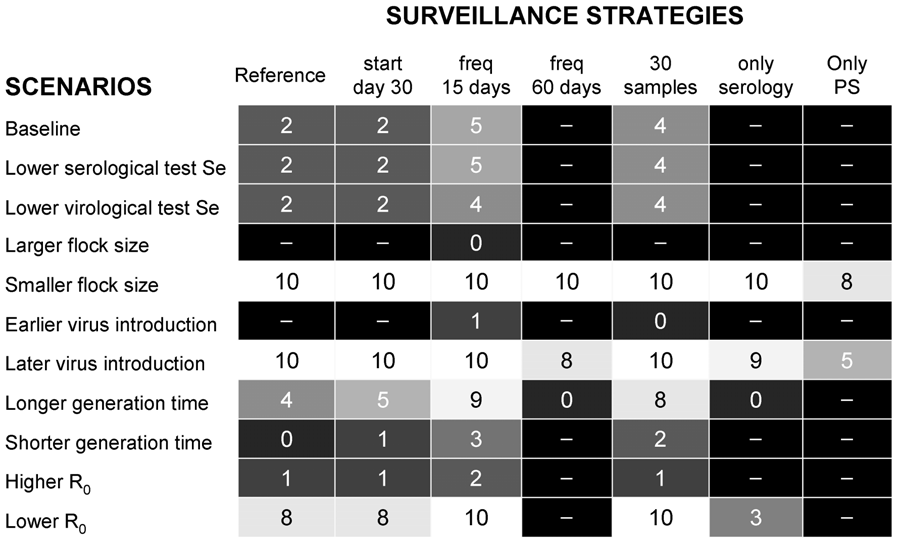
Effectiveness of outbreak detection. Grey-scale plot indicating the maximum number of days after positive sampling to efficiently stop the infection (i.e. last day at which R_h_<1) for alternative surveillance strategies under different scenarios. **Footnote**: The number reported in each cell represents the last day at which R_h_<1 for that specific combination of surveillance and scenario (light = better, dark = worse).

To better understand the effectiveness of the surveillance strategies under different scenarios, we determined the maximum number of days after detection day at which R_h_ was still below 1, which indicates the time available to prevent an epidemic (Figure 3). When considering the reference (or “baseline") scenario, to prevent an epidemic, between-farm transmission would need to be stopped by control measures within 2 days of the positive sampling. The use of imperfect diagnostic tests (i.e., lower test sensitivity) did not result in a significant worsening of the effectiveness of the surveillance strategies. When increasing the sampling frequency (once every 15 days), the maximum number of days allowed for preventing an epidemic increased to 5 (i.e., providing 3 more days to stop between-farm transmission). Increasing the sample size (30 birds) also increased the maximum number of days for preventing an epidemic (i.e., 4 days). By contrast, performing only passive surveillance made it impossible to detect infection in time to sufficiently reduce transmission to other farms (i.e., R_h_>1), except in the case of small farms or late virus introduction. Similarly, the use of only serological testing did not allow for extra time to set up control measures except in case of lower R_0_ or late virus introduction.

## Discussion

In this study, we evaluated diverse surveillance strategies for the purpose of early warning of LPAI epidemics, considering the actual epidemiological conditions during the Italian LPAI surveillance programme in 2000. Surveillance was only successful in preventing an epidemic if actions were taken within two days of sampling, which is rather unfeasible, given the waiting time to have the results from the laboratory. The sensitivity analysis showed that the surveillance strategies are not effective for larger farms or if the LPAI virus is introduced in the farm earlier or has a shorter generation time or a higher R_0_. In the last three scenarios, a high infectivity level is reached before detection (i.e., high AUC at detection), which results in an R_h_>1; on the other hand, under these circumstances the detection rate is higher (i.e., ∼80–90%, Figure 2.b) mostly due to the increase of the detection by passive surveillance. Furthermore, the surveillance strategies do not seem to be affected by the quality of the diagnostic test within the range examined (range based on van der Goot *et al.* [13]), although performing both serological and virological assays is important for increasing the sensitivity of active surveillance strategies. Given that preventive action must be taken as soon as possible, imperfect diagnostic tests which provide results quickly may be preferable to a higher-quality assay that takes longer.

The comparison of different sampling strategies showed that increasing the sampling frequency is the most effective means of improving the timeliness of detection (i.e., R_h_ is minimized) but that increasing the sample size increases the likelihood of detection (i.e. smaller outbreaks have a higher chance to be detected). This has also reported for the surveillance of other viral and bacterial infectious diseases, in particular, bovine herpesvirus I [14], *Mycobacterium bovis* [15], and *Salmonella* enteritidis [16]. However, it has been demonstrated that there is a limit to optimization by increasing the frequency and decreasing the sample size, given that if the size of the sample is too small, then the specificity of the surveillance decreases [16].

The reference scenario was the epidemiological situation at the beginning of the first Italian LPAI epidemic, when no compulsory biosecurity measures were in place. At present, commercial turkey farms must be managed according to strict biosecurity measures [17], which consist of physical and temporal barriers, cleaning, and disinfection. Given that such measures reduce both the risk of incursion of AI viruses in individual production units (i.e., bioexclusion) and the risk of outward transmission (i.e., biocontainment) [18], the current between-farm transmission may be lower than that in 2000, which could imply that current LPAI surveillance in Italy is more effective than indicated by our results.

We simulated passive surveillance using a threshold value, yet in reality passive surveillance depends on many factors, such as the virus strain, the infectious dose, the individual susceptibility of the farm (which may be enhanced, for instance, by concurrent infections with other pathogens), and the awareness of the farmer. However, our intention was to simulate a situation in which a certain percentage of outbreaks would be detected by passive surveillance, which is related to the efficiency with which infection spreads in the farm. Of the outbreaks simulated, about 26% were detected by passive surveillance (Table 5), which is consistent with the data from the 96 actual outbreaks (25%). However, in simulated outbreaks based on the 96 real farms (Table 2), 37% would have reached the threshold (i.e., if they had not been detected beforehand by active surveillance or had not terminated too early because of the end of the production cycle) (data not shown). This result is consistent with the estimated sensitivity of passive surveillance for LPAI in chickens reported by Alba et al. (2010) [8] using a scenario-tree approach (i.e., 36%, assuming a design prevalence at holding level of 5%).

Although we chose to focus our evaluation on LPAI in turkeys, we can speculate on how the surveillance strategies would perform in chickens. Chickens are known to be less susceptible to LPAI infection than turkeys [19], [20] and laboratory experiments have shown that bird-to-bird transmission of LPAI viruses in chickens can be low to moderate, depending on the virus strain [21], [22]. Furthermore, in densely populated poultry areas with both turkey and chicken farms, LPAI outbreaks have often occurred only on turkey farms, for example in Virginia in 2002 [23], in Italy in 2004 and 2005 [24], and in Germany in 2008 [25]. We can thus assume that both within- and between-farm transmission of LPAI infections are less efficient in chickens than in turkeys, as supported by the fact that, to date, no massive LPAI epidemics have been reported in chickens [26], [27]. However, a distinction should be made between broilers and layers, which have different rearing systems and lengths of production life. Although for layers passive surveillance based on decreases in egg production and feed intake has in some cases contributed to the prompt recognition of LPAI [28], the early detection of LPAI can be difficult to achieve in broilers unless many samples are tested very frequently.

Current EU legislation on the control of avian influenza focuses on early detection and prompt reaction in the event of an outbreak [3]; however, the primary goal of EU surveillance programmes for AI in poultry is to detect the annual presence of infections caused by the subtypes H5 and H7 of HPAI and LPAI [6]. Active surveillance programmes that include only one sampling event per production cycle probably result in missed or late detection of LPAI virus incursions in turkeys.

In conclusion, in this study, we tested a number of surveillance strategies that can be used for the early detection of LPAI infections, thus preventing major epidemics and the possibility of a virulence shift to HPAI. Early detection of LPAI outbreaks in turkeys can be achieved through the combination of passive and active surveillance. Passive surveillance may be quite effective when clear clinical signs are present, but for strains similar to those on which our model parameters were based (circulating in Italy in 2000–2005), active surveillance is needed as well. Concerning the sampling strategies for active surveillance, increasing the sampling frequency to once every 15 days leads to prompt detection, providing 5 days to react after taking a positive sample to prevent a major epidemic (R_h_<1). However, taking samples this frequently may not be sustainable in the long term, for both economical and practical reasons. Nonetheless, we deem the above mentioned strategy specifically suitable for cases in which either an LPAI virus has been recently introduced in a previously unaffected area or when the surveillance activities are performed in an area with a high risk of virus exposure. When no LPAI virus is circulating and there is no immediate risk of virus introduction, a less frequent sampling approach might be admitted, provided that the surveillance is intensified as soon as the first outbreak is detected. It would be useful to address such risk-based optimization of surveillance in a future study.
